# Prostate Cancer Scoring Index for Risk of Progression of Radioresistant Disease

**DOI:** 10.3390/jpm13050870

**Published:** 2023-05-22

**Authors:** Eleonora Cini Tesar, Ivana Mikolasevic, Iva Skocilic, Arnela Redjovic, Damir Vucinic, Jasna Marusic, Gordana Djordjevic

**Affiliations:** 1 Department of Radiotherapy and Oncology, UHC, 51000 Rijeka, Croatia; ivana.mikolasevic@gmail.com (I.M.); iskocilic@gmail.com (I.S.); redzovica@gmail.com (A.R.); damir.vucinic@gmail.com (D.V.); jasnaskrobonja@yahoo.com (J.M.); 2 Faculty of Medicine, University of Rijeka, 51000 Rijeka, Croatia; gordana.dordevic@medri.uniri.hr; 3 Department of Pathology, UHC, 51000 Rijeka, Croatia

**Keywords:** prostate cancer, immunohistochemistry, radiotherapy resistance, biomarkers, disease progression

## Abstract

Prostate cancer (Pca) is among the most common malignant diseases in men and the fourth leading cause of death worldwide. Surgery and radical radiotherapy (RT) remain the gold standard for the treatment of localized or locally advanced prostate cancer. The efficiency of radiotherapy treatment is limited by toxic side effects due to dose escalation. Cancer cells often develop radio-resistant mechanisms that are related to the DNA repair, inhibition of apoptosis or changes in cell cycle. Based on our earlier research on biomarkers that are involved in those cellular mechanisms (p53, bcl-2, NF-kb, Cripto-1 and Ki67 proliferation) and correlation with clinico-pathological parameters (age, PSA value, Gleason score, grade group, prognostic group), we created the numerical index for risk of tumor progression in patients with radioresistant tumors. For each of these parameters, the strength of association with disease progression was statistically assessed, and a specific number of points was assigned proportional to the strength of the correlation. Statistical analysis identified an optimal cut-off score of 22 or more as an indicator of significant risk for progression with a sensitivity of 91.7% and a specificity of 66.7%. The scoring system in the retrospective receiver operating characteristic analysis showed AUC of 0.82. The potential value of this scoring is the possibility of identifying patients with clinically significant radioresistant Pca.

## 1. Introduction

Prostate cancer is among the most common malignant diseases in men and is one of the leading health problems of elderly men [1]. Croatian Registry for Cancer ranked prostate cancer on the first place in terms of frequency and on the second place in terms of death from malignant diseases in men. The introduction of PSA tests in clinical practice helps us in the early detection of prostate cancer patients and has resulted in an increase in the number of patients with localized prostate cancer. Surgery and radiotherapy remain the gold standard for the treatment of localized or locally advanced prostate cancer [2]. The standard biomarkers that significantly predict the recurrence and progression of prostate cancer and choice of therapy are based on the validation of prostate-specific antigen (PSA) and pathological grade using Gleason score (GS) [3,4,5]. Gleason scores define the degree of differentiation of prostate cancer. Based on differentiation, a new prostate cancer grading system was developed during the 2014 International Society of Urological Pathology (ISUP) Consensus Conference. The new system assigns Grade Groups from 1 to 5, derived from the Gleason score, as follows: Grade Group 1—Gleason score ≤6; Grade Group 2—Gleason score 3 + 4 = 7; Grade Group 3—Gleason score 4 + 3 = 7; Grade Group 4—Gleason score 4 + 4 = 8; 3 + 5 = 8; 5 + 3 = 8; and Grade Group 5—Gleason score 9–10.

American Joint Committee on Cancer developed prognostic stage grouping. Prognostic Group 1 is defined with Gleason score of 6, PSA level less than 10 nanograms per milliliter and cT 1a-1c and cT2 stage. Prognostic Group 2 is defined with a Gleason score 3 + 4 = 7. This is Grade Group 2 or PSA level between 10 and 20 ng/mL and a T stage 1 and 2. Prognostic Group 3 has a Gleason score of 3 + 4 = 7 and a PSA level between 10 and 20 ng/mL and a T stage of 1 or 2 or a Gleason score 4 + 3 = 7 and a T stage of 1 or 2. Prognostic Group 4 has one of the following: Gleason score of 8 (this is Grade Group 4), PSA level higher than 20 ng/mL and T stage of 3. Prognostic Group 5 has two or more of the following: Gleason score 8, PSA level higher than 20 ng/mL, T stage of 3 or Gleason score 9 to 10. This is Grade Group 5 or T stage of 4.

Still, we have no tools to assess the clinically significant prostate cancer and determine the most appropriate and effective therapeutic strategy [6]. The result of radiotherapy in the treatment of prostate cancer varies; disease can stay indolent, or it can enter metastasis. The efficiency of radiotherapy treatment is limited by toxic side effects due to dose escalation. Moreover, cancer cells often develop radio-resistant mechanisms that are related to the DNA repair response, inhibition of apoptosis and changes in cell cycle. Biomarkers such as NF-kB, p53 and bcl-2 are activated by DNA damaging agents and may be responsible for reaction of the cell on radiotherapy treatment. The tumor suppressor p53 is a major regulator of cellular responses to DNA-damaging agents such as ionizing radiation. This transcription factor regulates cell-cycle control and checkpoints, cellular differentiation, apoptotic pathways, cellular senescence and angiogenesis [7]. Depending on the degree of DNA damage from ionizing radiation, p53 can promote cellular repair through cell-cycle arrest; p53 promotes apoptosis in cells with substantial radiation-induced damage [8,9,10]. Mutations in the gene encoding p53 occur in 4–60% of prostate cancers. Mutant p53 function permits mitosis before radiation-induced DNA damage is repaired, indicating that p53 may partly determine the cell’s sensitivity to damage induced by radiation therapy. Radiation Therapy Oncology Group studies 9202 and 8610 uncovered a correlation between p53 expression and radiotherapy outcome [11], but Incognito et al. did not find any such connection [12]. The majority of human prostate tumors overexpress Bcl-2, and we already know from the literature that overexpression is responsible for tumor resistance to radiotherapy and chemotherapy as a predominant antiapoptotic protein [13]. Moreover, it has been reported that Bcl-2 expression is associated with tumor progression and development of androgen-independent prostate cancer. NF-κB is a transcription factor that regulates the expression of genes linked to invasion, apoptosis, survival, inflammation, proliferation, angiogenesis, metastasis, chemoresistance, tumor cell transformation and radioresistance [14]. NF-κB may activate the expression of several genes or proteins that are involved in apoptotic regulation. The activity of the transcription factor NF-κB, along with many others, is enhanced in response to irradiation, leading to radiation resistance. Its inhibition, on the other hand, results in the radiosensitivity of Cap cell lines through increased apoptosis. One of the stem cell markers Cripto-1 is recognized as important in prostate cancer progression and a potential target for therapy. Cripto-1 is highly expressed in cancer stem cells in human prostate cell lines which are resistant to chemo and radiation therapy [15]. Our previous studies (unpublished data) evaluate and correlate the expression levels of p53, bcl-2, NF-kb, Cripto-1 and Ki67 proliferation index in prostate cancer (Pca) patients treated with radical radiotherapy and their association with clinicopathological profiles and disease outcomes. In the present study, we created a prognostic numerical index based on statistical analysis of retrospective data in combination with the level of immunoexpression of cellular proteins involved in the development of tumor radioresistance. For each of these parameters, the strength of association with disease progression was statistically assessed, and a specific number of points was assigned proportional to the strength of the correlation. Therefore, we were interested to investigate the scoring system that could help us to predict disease progression after radiotherapy treatment in our population of Pca patients.

## 2. Patients and Methods

### 2.1. Patients and Tissue Samples

In this retrospective analysis, we analyzed 92 patients at the Department of Radiotherapy and Oncology, Rijeka University Hospital Center, Rijeka, Croatia, and the Department of Pathology, Medical faculty, University of Rijeka, Croatia, between November 1998 and December 2010. Patients were treated at the Department of Radiotherapy and Oncology with radical radiotherapy (total dose 64 Gy) and followed for at least 48 months. Inclusion criteria were age above 18 years, patients that were treated by radical radiotherapy with locally advanced disease, patients that had pathohistological sample that was adequate for immunohistochemistry analysis and those with complete medical data. Out of 92 initially analyzed patients we excluded 23 patients due to incomplete medical data or inadequate pathohistological sample or metastatic disease at the time of diagnosis.

In all cases, pathology indicated a diagnosis of prostate acinar adenocarcinoma. For the purposes of this study, 69 patients with all data were selected. Clinicopathological characteristics of the patients, collected from medical records, are summarized in Table 1.

### 2.2. Radiotherapy

A total of 69 men received external beam radiotherapy for clinically localized prostate cancer. The daily fractionation dose 1.8 Gy and the total dose 63 Gy were applied in each case on therapeutic machine Siemens Oncor Expression. At the Department of Radiotherapy and Oncology, Rijeka University Hospital Center, Rijeka, between November 1998 and December 2010, our technical capabilities of devices were the same, so we performed conventional, 2D or box technique. The beginning of 3D radiotherapy in our institution was only in 2012 when we obtained a CT simulator. A box technique was used because it is easy to plan with a conventional simulator and dose conformity produced to the target. Some analysis showed that this technique produces planning results comparable to those achieved with more complex techniques such as 3D and IMRT. To determine the target volume in 2D radiotherapy, a 2D X-ray device called a diascope was used, and the bone structure defined the boundaries of individual fields. For large field treatment, the superior border of the pelvic field was usually set at the level of the mid-sacroiliac joints, and the inferior border was set at the bottom of the ischial tuberosities. Lateral borders on the anterior/posterior and posterior/anterior fields are set 1.5 to 2.0 cm lateral to the pelvic brim, and the posterior border is set at the S2/S3 interspace. Anteriorly, the field edge is at the front edge of the pubic symphysis. The field edges for the comedown and small pelvic field treatment extend superiorly to the top of the acetabulum and laterally include two-thirds of the obturator foramen. Dose distributions for conventional treatment are typically generated in a single plane, and the dose is prescribed at the isocenter and normalized at the 100% isodose line.

The conventional method of radiotherapy was used in the early 1990s, but its application limited the administration of higher doses of radiation to the prostate due to its toxicity to the risk organs. With the introduction of 3D conformal radiation planning, the number of side effects was reduced, the dose of the target volume were raised, and better disease control was achieved. Pollack et al. [16] conducted a comparison study of the conventional and 3D treatment plans and revealed that the volume of doses to the rectum (over 60 Gy) was equivalent but significantly less than to the bladder, which was in the high dose volume in the 3D-CRT plans. There were no differences between these two modalities in terms of acute toxicity or early biochemical response. Lower standard doses might still be effective if combined with androgen deprivation therapy; several randomized studies demonstrated a significant advantage in terms of specific or overall survival by combining ADT to RT at lower doses (63–70 Gy) than those currently considered as standards (>75 Gy).

### 2.3. Sample Preparation and Immunohistochemistry

**Figure 1:**
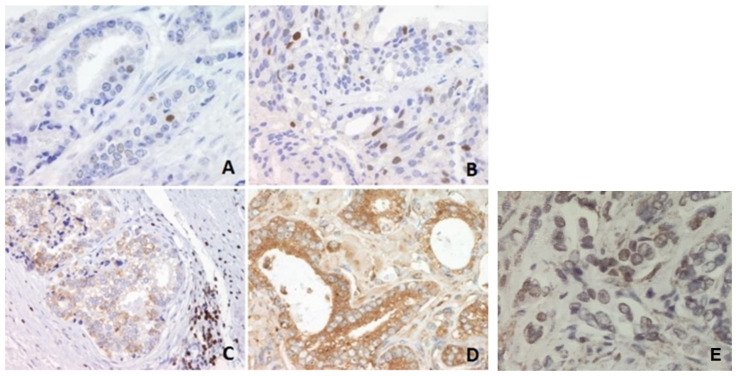
Immunohistochemical analysis of p53, Bcl-2 and NF-κB protein levels in prostate carcinoma tissues. (**A**) Infrequent nuclear immunostaining of p53 in a better-differentiated tumor, magnification 400×. (**B**) More frequent nuclear immunostaining of p53 in a higher-grade tumor, magnification 400×. (**C**) Granular cytoplasmic reaction of NF-κB, magnification 200×. (**D**) Granular cytoplasmic reaction of NF-κB., magnification 400×. (**E**) Cripto-1 nuclear immunopositivity (magnification 200×).

Tissue microarrays were constructed. For each case, formalin-fixed, paraffin-embedded tumor tissue was sampled in triplicate; morphologically normal prostate tissues served as controls. The resulting blocks were cut into 5-µm sections for immunohistochemistry. Heat-induced pretreatment was used for retrieval of p53 antigen. Slides were placed in 10 mM citrate buffer [pH 6.0] and heated in a microwave for 10 min at 800 W and then for another 15 min at 450 W. For Bcl-2 and NF-κB, slides were treated with Tris/ethylenediaminetetraacetic acid [pH 9] at 98 °C for 15 min. After antigen retrieval, the DakoEn/Vision +/HRP Kit was used to visualize p53, Bcl-2 and NF-κB protein. Slides were incubated separately with anti-p53 mouse monoclonal antibody (Clone: DO-7, DakoCytomation, Glostrup, Denmark), anti-Bcl-2 mouse monoclonal antibody (Clone: M 0887, DakoCytomation, Glostrup, Denmark) and anti-NFκBp65 mouse monoclonal antibody (Clone: F-6 Santa Cruz Biotechnology, Dallas, TX, USA). For determination of proliferative activity in tumor cells (Ki-67 index) we used a monoclonal antibody (clone MIB-1, dilution 1:50, Dakocytomation, Glostr-up, Denmark). The immunoexpression of the Crypto-1 molecule was determined using a rabbit polyclonal antibody against human CR-1 (cat. no., SAB1306280; Sigma-Aldrich; St. Louis, MO, USA, dilution, 1:80). The levels of each protein were determined using a computer assisted IHC quantification on xy cases. All slides stained with xy were scanned and analyzed using Alphelys Spot Browser 2 integrated system, consisting of software controlled (Spot Browser 2.4.4, Alphelys, Plaisir, France) motorized stage microscope (Eclipse 50i, Nikon, Tokyo, Japan) mounted digital camera (1360 × 1024 resolution, 24-bit, CFW-1310C, Microvision, Lisses, France). Slides without primary antibodies served as negative controls. Nuclear staining was p53 and NF-κB. Cytoplasmatic staining was bcl-2. Nuclear, cytoplasmatic and membranous staining was Crypto. We compared the immune expression percentage of nuclear, cytoplasmic and membranous positivity and clinicopathological factors such as PSA, Gleason score, grade and prognostic groups [17] with biochemical progression and overall survival (Figure 1).

### 2.4. Statistical Analysis

For each of the parameters mentioned, the strength of association with progression of cancer was estimated whereby we defined the progression as PSA relapse. When designing the numerical index, we included parameters already known as prognostic such as PSA value prior the therapy, Gleason score of biopsy or prostatectomy, proliferation index and mentioned biomarkers involved in radioresistance. Complete data were available for 69 patients, which were then statistically analyzed. A specific number of points was assigned to each of these parameters, proportional to the strength of the statistical association. A statistical association between nominal variables was measured by Cramer’s V coefficient, whereby a minimum coefficient value of 0.15 was required. For the scoring system, coefficient values were divided by a minimum value and rounded to the nearest integer. Sensitivity curve analysis (receiver operating characteristic curves, ROC curves) and Youden’s index J were used to determine the sensitivity and specificity of a particular test and the optimal cut-off value. Cut-off values were optimized using the Youden index maximization criterion. For all analyses, values of *p* < 0.05 were considered statistically significant, and values of *p* < 0.1 were considered weakly statistically significant.

## 3. Results

### Clinicopathological Data

In total, 69 prostate cancer patients that have been treated with radical radiotherapy at the Department of Radiotherapy and Oncology were enrolled in the study. Our samples consisted of 51% low-grade tumors (Gleason score ≤ 6) and 49% high-grade tumors (Gleason score ≥ 8; Table 1). The participants’ baseline characteristics are shown in Table 1.

**Figure 2:**
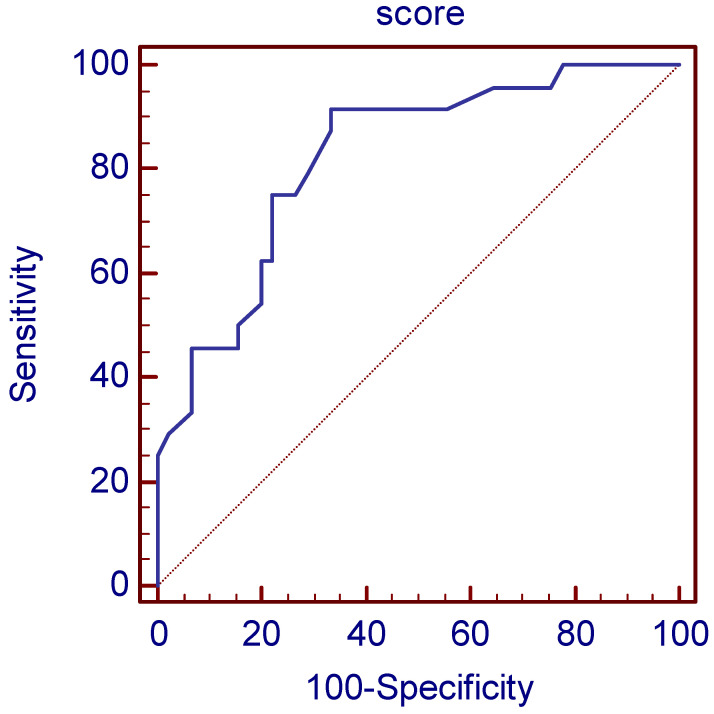
The scoring system in the retrospective analysis of the operating characteristics of the receiver showed an AUC of 0.82. (*p* < 0.0001).

In the numerical index, we present relevant parameters that influence tumor radioresistance associated with disease progression. The corresponding number of points assigned in the numerical index is shown in Table 2. Statistical analysis identified an optimal cut-off score of >21 or more as an indicator of significant risk for progression with a sensitivity of 91.7% and a specificity of 66.7%. We have assumed that a minimum value of 0.1 for Cramer’s V coefficient proves the presence of an association. The scoring system in the retrospective analysis of the operating characteristics of the receiver showed an AUC of 0.81 with the least (*p* < 0.1) (Figure 2). Statistical association between nominal variables was measured by Cramer’s V coefficient, whereby a minimum coefficient value of 0.1 was required. For the scoring system, the coefficient values were divided by a minimum value and rounded to the nearest integer. The cutoff value of the scoring system was determined by optimization of sensitivity and specificity based on the Youden index together with classical receiver operating characteristic analysis.

The points for each parameter, as we can see in Table 3, in the scoring system are determined as follows. First, the strength of the relationship between each parameter was determined and expressed by Cramer’s V coefficient. The value with the smallest coefficient greater than 0.1 was considered the baseline value, and for all other parameters with a coefficient greater than the baseline value, the ratio of their coefficient to the baseline value was determined. Parameters with a Cramer’s V coefficient less than 0.1 were assigned a ratio value of 0. To obtain points expressed in whole numbers, the ratios were multiplied by the common denominator and rounded to an integer value.

The cut-off value was determined using ROC analysis, with the Youden index calculated for each cut-off value. The Youden index is defined as sensitivity + specificity −1. The cut-off value with the highest Youden index is considered the optimal cut-off value. This score has an AUC of 0.82 (0.71–0.90), and with a cut-off value of >21, it gives a sensitivity of 91.67% (73.0–99.0%) and a specificity of 66.67% (51.0–80.0%), while PPV was 59.46% (42.10–75.25%) and NPV 93.75% (79.19–99.23%) for detection of high-risk patients for disease progression. Of the 69 analyzed patients, 24 patients had a PSA relapse, and 45 did not. RSA relapse was predicted in 37 patients using the score. There were 22 true positives and 2 false negatives with 15 false positives and 30 true negatives (Figure 2).

## 4. Discussion

An aging population and the modern lifestyle have contributed to significant increases in the incidence of prostate cancer. Most men diagnosed with prostate cancer do not die from the disease, but with 10–25% of prostate-cancer patients dying of metastatic disease, it remains the second leading cause of cancer death in men [1]. The standard treatment modalities for organ-confined prostate cancer are prostatectomy and radiotherapy. However, not all patients benefit from radiotherapy, and the individual response to radiotherapy varies. Nearly 30% of patients undergoing radical radiotherapy for clinically localized disease still experience disease progression. Patient outcomes are unpredictable, and survival varies greatly. Recently, ProtecT reported baseline clinico-pathological characteristics of men with localized Pca and concluded that the lack of reliable prognostic biomarkers hinders the formulation of accurate prognoses, and disease progression cannot be predicted reliably [6]. Prostate cancer is heterogeneous, which has implications for the selection of treatment that would be more optimal if there were prognostic post-biopsy or postoperative biomarkers. Today, prognostic molecular biomarkers such as the cell cycle progression score (CCP) are in use [17]. The prognostic signature of RNA expression levels of 31 CCP genes is an important clinical tool for standardizing risk factors for disease progression, especially in combination with clinical progression scores [17]. In our numerical index, we present the relevant parameters that influence tumor radioresistance associated with disease progression, which has been proven through previous research in the literature.

Age as a well-known risk factor for the development of PC is again in the focus of prognostic studies. Japanese authors recently took age as specific prognostic factor in biochemical recurrence in patients treated with brachytherapy and concluded that younger patients under the age of 60 achieved long-term cancer control, without major side effects of RT [18]. Göteborg-1 Prostate Cancer Screening Trial finds an increased risk of clinically significant PC with aging and questions the optimal age for screening and early detection of PC [19]. The inclusion of prognostic and predictive biomarkers that influence the outcome of radiotherapy such as in our study is also necessary in the timely recognition of disease progression.

It has been shown that radiotherapy could also participate in the selection and enrichment of PCA carcinoma stem-like cells (CSCs), which contribute to radioresistance and the initiation of metastases through the activation of epithelial-mesenchymal transition (EMT) [20,21,22]. One of the stem cell markers Cripto-1 is recognized as important in prostate cancer progression and potential target for immunotherapy. Cripto-1 is highly expressed in cancer stem cells in human prostate cell lines which are resistant to chemo and radiation therapy and is also of interest for our study and numerical index. Univariate and multivariate analysis by Liu et al. showed that high immunoexpression of CR-1 in the tumor was significantly associated with shorter survival without biochemical relapse [23].

The largest study to evaluate expression of 11 parameters in 677 prostate-cancer patients treated with radiotherapy was that of the Radiation Therapy Oncology Group. Abnormal p53 levels were noted in 22% of these cases and were associated with cause-specific survival (*p* = 0.014) and risk of distant metastasis (*p* = 0.013) [24].

Another large study was that of Grigon et al. [25], who assessed the prognostic value of p53 overexpression in tumors from 471 patients with prostate cancer who were treated with either external-beam radiation therapy alone or with total androgen blockade. Statistically significant associations were uncovered between abnormal p53 protein expression and increased incidence of distant metastases, decreased overall survival, and decreased progression-free survival. D’Amico et al. reported a significant connection between p53 expression and disease recurrence in men treated with radiation [26]. Abnormalities in the expression levels of Bcl-2 and Bcl-2-associated X protein are consistently associated with increased failure after external beam radiotherapy for prostate carcinoma [16]. Smaller studies previously evaluated the expression of p53, Bcl-2 and Ki-67 [27,28,29]. Lee et al. developed a scoring system that provides an estimation risk of biochemical failure after salvage radiotherapy, based on PSA, pathological tumor stage, Gleason score and surgical margin status with a c-index of 0.66 [30].

The opposite is the research of Freedland, Gerber, Reid et al. who focused just on RNA signature without clinical pathological parameters. They found cell cycle progression score, a prognostic RNA signature based on the average expression level of 31 CCP genes that has been shown to predict biochemical recurrence (BCR) after primary external beam radiotherapy [31].

Our unpublished results showed usefulness of three biomarkers (p53, bcl2 and NfKB) that can together significantly predict progression of the disease. An increased value of each of the given parameters means a decrease in the probability that a person will endure 5 years without progression by about 1/0.3 = 3.3 times. It is significant that the exclusion of any of the three parameters impairs the predictive value of the model. We also found correlation of cytoplasmic immunoexpression with the stage and of membranous with PSA level, while lower nuclear Cripto-1 positivity showed a weak association with the occurrence of disease progression.

According to our best knowledge, in this study we proposed for the first time a prognostic numerical index based on statistical analysis of retrospective data in combination with the level of immunoexpression of cellular proteins involved in the development of tumor radioresistance. The presented numerical index indicates that radioresistant patients with a score higher than 21 will be candidates for disease progression, which is the main message of this study. Actually, a score below 21 has an excellent discrimination that with a high degree of certainty, recognizing those patients that will not have disease progression. Thus, our index is an excellent marker that can identify those patients that will not have disease progression if the score is 21 or less. However, our study has some limitations, such as the relatively small number of analyzed cases and retrospective design. Therefore, further larger studies that will prospectively test this index in patients with localized prostate cancer are needed.

## Figures and Tables

**Table 1:** Clinical and pathological characteristics of patients with prostate cancer treated with radiotherapy.

Characteristic	
Age of patients (years, mean ± SD)	65.08 ± 6.93
Level of serum PSA at the time of the dg of Pca (ng/mL, mean ± SD)	24.11 ± 29.42
Pathologic stage (%)	
cT1	52.17
cT2	4.35
cT3	43.48
Gleason score (%)	
≤6	50.72
≥8	49.28
Ki 67 (mean ± SD)	8.78 ± 14.08

PSA, prostate specific antigen; SD, standard deviation.

**Table 2:** Relevant parameters that influence tumor radioresistance associated with disease progression.

Parameter	Sensitivity (%) (95% CI)	Specificity (%) (95% CI)	AUC (95% CI)
Age (years) (>72)	24.24 (11.1–42.3)	92.31 (81.5–97.9)	0.51 (0.39–0.62)
PSA (nmol/L) (>8.3)	88.89 (73.9–96.9)	60.00 (45.2–73.6)	0.71 (0.60–0.80)
Gleason score (≥7)	72.22 (54.8–85.8)	59.26 (45.0–72.4)	0.71 (0.60–0.08)
grade group 1	25.00 (12.1–42.2)	42.59 (29.2–56.8)	0.34 (0.24–0.45)
grade group 2	44.00 (24.4–65.1)	61.54 (48.6–73.3)	0.53 (0.42–063)
grade group 3	66.67 (9.4–99.2)	60.92 (49.9–71.2)	0.64 (0.53–0.74)
grade group 4	44.44 (13.7–78.8)	60.49 (49.0–71.2)	0.53 (0.42–0.63)
grade group 5	76.92 (46.2–95.0)	66.23 (54.6–76.6)	0.72 (0.61–0.81)
prognostic group 1	5.56 (0.7–18.7)	82.69 (69.7–91.8)	0.44 (0.34–0.55)
prognostic group 2	25.00 (12.1–42.2)	75.00 (61.1–86.0)	0.50 (0.39–0.61)
prognostic group 3	2.78 (0.07–14.5)	98.08 (89.7–100.0)	0.50 (0.40–0.61)
prognostic group 4	22.22 (10.1–39.1)	55.77 (41.3–69.5)	0.39 (0.29–0.50)
prognostic group 5	72.73 (49.8–89.3)	69.70 (57.1–80.4)	0.71 (0.61–0.80)
cripto1 (>0.25)	64.00 (42.5–82.0)	48.98 (34.4–63.7)	0.53 (0.41–0.65)
p53 (>0.169)	22.22 (10.1–39.2)	94.44 (84.6–98.8)	0.55 (0.44–0.65)
bcl2 (>0.1256)	35.29 (19.7–53.5)	83.33 (70.7–92.1)	0.58 (0.47–0.69)
NFKb (>0.309)	79.41 (62.1–91.3)	47.17 (33.3–61.4)	0.62 (0.52–0.73)
Ki67 (>0.0445)	60.00 (42.1–76.1)	75.93 (62.4–86.5)	0.69 (0.58–0.78)

Note: Cut-off values are optimized using the Youden index maximization criterion; PSA, prostate specific antigen.

**Table 3:** Score formation, an optimal cut-off score of >21 or more as an indicator of significant risk for progression.

Parameter	Cramer’s V Coefficient	Cramer’s V Ratio	Points
Age (>72)	0.2316	2.29	9
PSA (>8.3)	0.4933	4.88	20
Gleason score (≥7)	0.3091	3.06	12
grade group 1	−0.3195	−3.16	−13
grade group 2	0.0506	0	0
grade group 3	0.1011	1	4
grade group 4	0.0302	0	0
grade group 5	0.3097	3.06	12
prognostic group 1	−0.1747	−1.73	−7
prognostic group 2	0.0000	0	0
prognostic group 3	0.0282	0	0
prognostic group 4	−0.2265	−2.24	−9
prognostic group 5	0.3736	3.7	15
cripto1 (>0.25)	0.1235	1.22	5
p53 (>0.169)	0.2493	2.47	10
bcl2 (>0.1256)	0.2128	2.10	8
NFKb (>0.309)	0.2690	2.66	11
Ki67 (>0.0445)	0.3612	3.57	14

PSA, prostate specific antigen.

## Data Availability

Data are available upon request.
